# Qualitative assessment of knowledge and attitudes towards cervical cancer screening among male Latino immigrants in Houston, Texas

**DOI:** 10.1186/s12905-020-01006-5

**Published:** 2020-07-06

**Authors:** Susan H. Read, Ivan Valverde, Jane R. Montealegre, Thomas J. Rutherford, Matthew L. Anderson

**Affiliations:** 1 Division of Gynecologic Oncology, Department of Obstetrics & Gynecology, Morsani College of Medicine, University of South Florida, 12901 Bruce B. Downs Boulevard, MDC 2040A, Tampa, FL 33612 USA; 2 H. Lee Moffitt Cancer Center and Research Institute, Tampa, FL 33612 USA; 3 Houston Independent School District, Houston, TX USA; 4 Department of Pediatrics and Dan L. Duncan Comprehensive Cancer Center, Baylor College of Medicine, Houston, TX 77030 USA

**Keywords:** Cervical cancer screening, Male partners, Cancer prevention, Health behaviors

## Abstract

**Background:**

Male spouses and partners play an important role in determining a woman’s willingness to participate in cervical cancer screening. However, the attitudes and behaviors by which they influence a woman’s decision to undergo Pap testing remain poorly understood.

**Methods:**

A series of semi-structured, qualitative interviews were conducted in Spanish with 19 recent Latino immigrants in Houston, Texas. The interview format was designed to establish each individual’s pattern of engagement with the United States healthcare system, assess baseline knowledge of cervical cancer screening and evaluate attitudes and patterns of communication with their female partners regarding health care. Interview questions were constructed using principles of the Theory of Reasoned Action. All interviews were conducted in Spanish. After translation, responses were coded and scored with the goal of identifying themes and key observations.

**Results:**

Most subjects reported few, if any, interactions with the healthcare system since their arrival in the United States. Although most participants reported being aware that women should be seen by their doctors regularly, fewer than half could clearly indicate the purpose of a Pap test or could state with certainty the last time their female partner had undergone screening. Multiple subjects expressed a general distrust of the health care system and concern for its costs. Approximately half of subjects reported that they accompanied their female partner to the health care provider’s office and none of the participants reported that they were present in examination rooms at the time their partner underwent screening. Multiple participants endorsed that there may be some concerns within their community regarding women receiving frequent gynecologic care and distrust of the healthcare system. Almost all interviewed subjects stated that while they would allow their female partners to see male physicians, they also expressed the opinion that other men might be uncomfortable with this and that women would likely be more comfortable with female physicians.

**Conclusions:**

Strategies to enhance knowledge of HPV and cancer screening and improve trust in the health care system among male spouses or partners should be explored with the goal of promoting cervical cancer screening among immigrant Latinx populations.

## Background

Invasive carcinoma of the uterine cervix is the fourth most common cancer diagnosed in women worldwide. The World Health Organization (WHO) estimates that 570,000 new cases of cervical cancer were diagnosed in 2018 and that 311,000 women died from this disease [1]. In large part, the high frequency at which cervical cancer is diagnosed in many parts of the world reflects a lack of access to preventive health services that include screening and vaccination for human papillomavirus (HPV)-related illnesses. However, even in the United States (U.S.), cervical cancer and other HPV-related illnesses remain a significant public health issue. The American Cancer Society (ACS) estimates that 13,170 new cases of cervical cancer and 4250 cervix cancer-related deaths occurred in the United States (U.S.) in 2019 [2]. Of note, the incidence of cervical cancer in the U.S. is significantly higher among Hispanic, American Indian, and non-Hispanic African American patients than their non-Hispanic White and Asian counterparts [2]. Hispanic women, particularly those who born outside the U.S., are generally diagnosed with cervical cancer at later stages, and experience greater mortality from this disease than others [3, 4]. Multiple factors potentially account for this health disparity, including lower rates of cervical cancer screening among recent immigrants, difficulty accessing appropriate care, language barriers and rates of acculturation [5, 6].

It is now well established that most cervical cancers arise as a result of prior infection with specific, high-risk genotypes of HPV. Due to the lengthy interval between incident infection and the development of invasive disease, cervical cancer is preventable with appropriate population-based screening and, more recently, vaccination against HPV infection [1]. HPV and cervical cancer screening may be performed by one of two methods. The first is by the cytologic evaluation of cells collected from a woman’s cervix. This test, also known as a “Papanicolaou (Pap) smear”, has technically evolved since its initial development more than 70 years ago, but has proven highly effective at reducing the incidence of cervical cancer when used as the basis for population-based screening. More recently, multiple prospective clinical studies have shown that use of molecular profiling to evaluate specimens for evidence of active HPV infection can be used to improve both the sensitivity and specificity of traditional cervicovaginal cytology for high grade cervical pre-cancers. HPV testing is now frequently used in developed countries. However, access to these tests can remain a challenge, particularly in low resource settings and developing countries [7]. Most recently, a high-risk HPV (HR HPV) test that may be self-collected has been approved by the FDA. Self-testing may represent a more feasible screening option for women who are unable to participate in more traditional approaches to cervical cancer screening [8].

Unfortunately, population-based estimates indicate that only about 80% of age-eligible women in the U.S. are compliant with current recommendations for cervical cancer screening [9]. Despite the availability of HPV vaccines that have been shown to reduce the incidence of cervical cancer precursors, vaccine uptake and rates of completion remain a challenge within the U.S. [10] Furthermore, rates of HPV vaccination remain uneven across many parts of the world. Therefore, efforts to ensure effective cervical cancer screening remain important. Within the U.S., Hispanic women are less likely to receive Pap smears than other ethnic groups, even after controlling for barriers to care [11]. Furthermore, several recent studies have found that a significant proportion of the women non-compliant with screening report that they have never been screened despite regular visits to their primary care provider for other clinical indications [12–14]. Thus, efforts to address barriers to cervical cancer screening, particularly among high risk populations, will remain important for the foreseeable future.

Multiple factors potentially contribute to the lower rates of cervical cancer screening observed among Hispanic women residing in the U.S. Knowledge of HPV and cervical cancer is generally lower among Hispanic women than non-Hispanic Whites [15], and is particularly low among recent immigrants [16]. In developing countries and in areas of the U.S. with large immigrant populations, knowledge regarding HPV and its relationship to cancers, including cervical cancer, is often lacking [17]. For example, a recent survey conducted in Cordoba, Argentina found that only 62.1% of participants were aware of the relationship between HPV and cervical cancer, despite the fact that many of the participants were medical students, dental students, or graduate students in the biologic sciences [18].

Among Hispanic populations, there is some evidence from studies both in the U.S. and abroad that a male spouse or partner attitudes may influence women’s decision to participate in cervical cancer screening. For example, two studies conducted among a predominantly Latino population of medically underserved women in Houston, Texas recently identified a male spouse or partner as one of the three primary factors driving a woman’s decision to have a Pap smear [14, 19]. Knowledge of HPV and cervical cancer among men is generally low, and is even lower among Hispanic versus non-Hispanic White men [20], which may influence male attitudes toward cervical cancer screening [21]. However, contemporary attitudes and/or patterns of interactions by which male partners and spouses influence a women’s decision to participate in cervical cancer screening are poorly understood.

Here, we undertook an exploratory qualitative study with the goal of gaining insight into patterns of engagement among recent male Latino immigrants with the U.S. healthcare system, their knowledge of cervical cancer, and patterns of communication and preferences for care among their female partners. Our fundamental objective was to develop hypotheses that could be tested and used to reconcile observations that male partners influence a woman’s decision to undergo screening.

## Methods

### Ethical approval

Permission to perform a qualitative survey study was obtained from the Institutional Review Boards for both Baylor College of Medicine (H-40770) and the University of South Florida (Pro000400706).

### Survey construction

A format for conducting semi-structured interviews was developed and vetted by local content experts knowledgeable about HPV, women’s health and qualitative research. The interview format was designed to establish each subject’s pattern of engagement with the United States (U.S.) healthcare system, assess baseline knowledge of cervical cancer screening and evaluate attitudes and patterns of communication with their female partners regarding health care. Interview questions were constructed using principles of the Theory of Reasoned Action [22]. Content of these questions were reviewed by local content experts knowledgeable about women’s health and health disparities.

### Subject interviews

Subjects (*n* = 19) were enrolled by approaching individuals (*n* = 15) at approved community centers (*n* = 4). These sites were selected based on a) their location in neighborhoods known to be predominantly populated by immigrant communities in Houston, Texas and b) their ability to provide access to a room where interviews could be conducted in private. In addition to obtaining regulatory approval from the host academic institutions, permission to engage in study activities was obtained from management of each community center prior to approaching potential subjects. A smaller number of additional subjects (*n* = 4) were also recruited at the Mexican Consulate in Houston, Texas after obtaining permission from the Consul General for the Government of Mexico.

A trained interviewer approached potential participants and invited each individual to participate in a brief interview. Potential subjects were then screened to determine whether they met enrollment criteria. Participants were deemed to be eligible if they were primarily Spanish-speaking males, had been born outside the United States, were age > 18 years and self-reported that they were currently involved in a relationship with a female partner for > 1 year. Participants did not need to be married to their partners. Potential participants were verbally consented to participate in this study without restrictions. All interviews were conducted by a researcher fluent in Spanish, who used planned survey questions as a starting point with each participant to explore key subject content targeted for these interviews. Due to privacy concerns and potential impact on subjects’ willingness to participate, no other demographic data was routinely collected. Thus, specific data documenting country of origin, number of years living in Houston, Texas or the United States, educational background or religious beliefs are not available for analysis. At the completion of each interview, subjects were offered a $25 gift card for their participation. The number of subjects enrolled in this study was determined by its “point of saturation,” which was defined as the failure to identify new themes and/or substantively distinct responses for at least 5 consecutive interviews.

### Data abstraction

All interviews were digitally recorded, after which, they were transcribed and translated in its entirety by a validated, third party translation service (Production Transcripts, Inc., Glendale, CA). All transcripts and translations were tagged with the unique study identifier assigned to each subject at the time they complete their interview.

### Data analysis

Thematic analysis was used to identify emergent themes from the transcripts as previously described [23] Each transcript was initially closely read and re-read by each author so that each investigator could become familiar with the data and identify potential themes. The investigator team then meet to discuss potential themes. Each transcript was then manually coded by a single investigator (SR). No qualitative analysis software was utilized. An iterative process of collapsing initial codes into emergent themes and sub-themes. Particular consideration was given to retaining the diversity of the initial codes while producing over-arching subthemes. The themes and subthemes identified by these analyses were then reviewed by all investigators to establish consensus in their naming, definition, and contribution to the main research question of how male partners influence a woman’s decision to undergo screening. Themes that were considered by all investigators to make meaningful contribution to the research question were selected for refinement. Subject quotes congruent with the overarching themes were selected by one of the investigators (SR) and subsequently discussed among all investigators. The relevance of each quote to the overarching themes and central research question was agreed upon prior to inclusion in the final manuscript. Each quote included for publication is labeled with the study identifier assigned to subject. Responses falling within a particular theme or sub-theme were reported as percentages in order to provide some sense of the frequency at which they were reported by participants.

## Results

A total of 19 men agreed to participate and were interviewed. In two interviews, the participants had regional accents and audio quality for initial portions of the interview was poor. As a result, parts of these two interviews were unable to be translated in their entirety. All subjects reported that they had immigrated to the United States from Spanish-speaking countries in North, Central, or South America. All participants were primarily Spanish-speaking.

### Patterns of subject interaction with the healthcare system

Less than half of the participants indicated that they saw a doctor regularly themselves, and almost all of these saw their doctor every 6 to 12 months. Only one participant reported seeing a physician more frequently, secondary to poorly controlled diabetes. Those who saw a physician regularly almost all cited chronic conditions as the reason for their visits. Chronic illnesses described by subjects included diabetes, chronic obstructive pulmonary disease, and back pain. Participants who reported only going to the doctor occasionally typically reported doing so in response to an acute issue, such as an accident.

Interviewer: Tell me, how often do you go to the doctor?

Subject 12: Often, no. In 2007, I fell… In the [past] 10 years, I have been to a checkup

three times.

### Knowledge of female partner healthcare

Most subjects reported that their female partners saw a gynecologist regularly for Pap smears and mammograms, though one participant (Subject #8) stated that his wife had not seen a physician since her last pregnancy many years earlier. However, the same participant mentioned that he felt it was very easy and acceptable for his wife to be seen by a gynecologist regularly for preventative care visits, as she had insurance coverage. He indicated that for someone without the same insurance, preventative care would be more difficult to access. Another participant (Subject #9) reported that he and his wife were unable to see a physician as regularly as they had in their home country, secondary to lack of sufficient funds or insurance. An additional subject mentioned that his wife had been having abdominal pain for which she had been seen in the emergency room, but that they could not afford to be seen for regular clinic visits, leading them to try use of “natural medicine”.

Interviewer: Do you know if [your wife] has checkups?

Subject 16: Regularly, she hasn’t gone. She went to the hospital like about 5 months

ago because of pain she has in her belly. And she was having that pain and I had to take her to the emergency room at the hospital. But they [said] that she didn’t have anything... but the pain continues, and we’re just trying to treat it with like natural medicine which you don’t tell people this and that.

### Knowledge of cervical Cancer screening

Most subjects reported that they had heard of a Pap smear. However, misconceptions about the nature and purpose of this test were commonly observed. For example, one participant was under the impression that a Pap smear also screened for breast cancer, and one admitted that while he had heard of a Pap smear he was unaware of what it screened for.

Interviewer: ...do [you] know what a Pap test is, then?

Subject 2: Yes, it’s a women’s exam for checking cancer, and it has to do, at minimum,

at least what we talked about (cervical cancer), the same with the breasts, too.

Interviewer: So do you know if your wife does Pap tests?

Subject 13: I think that… she did the Pap test, a year ago now.

Interviewer: What do you know about the Pap test?

Subject 13: I don’t know exactly what it is, but I’m sure because of what my mom also

told me to tell my wife to do a Pap test, because they clean the... womb or something like that, but, no, I don’t know exactly what it is.

Only about half of participants who responded to the question (53%, or 42% of overall participants) reported that they had heard of cervical or uterine cancer. Only one participant reported that he was aware that cervical cancer could be the result of a male partner with an infection. In discussing their sources of information regarding cervical cancer, a small number of participants mentioned seeing television ads, and one mentioned reading pamphlets and other literature.

Interviewer: What do you know about uterine cancer or cervical cancer? Have you

heard about it before?

Subject 1: I’ve heard, but I won’t say, because I don’t remember … [I heard] on

television.

Interviewer: Do you know something about cervical cancer?

Subject 2: Yeah, it’s mainly transmitted by guys. It doesn’t have much to do with hygiene, especially the penis... The man thinks that the one he has, the woman is the one who does it and whatever, and that’s not it. It’s not like that. That goes hand in hand with sexual relations, after all. They’re involved... We take literature and we see the... pamphlets that they have there.

### Communication between partners

The majority of subjects interviewed reported that they discussed women’s health issues with their partner, at least occasionally. It was fairly common for participants to report that they went to the doctor with their partner regularly; however, when questioned more closely, they had often only been once in the past three to five years, or had only accompanied their partner when she was pregnant. One participant mentioned that he had been able to accompany his partner to the doctor much more frequently in their home country, but that the demands of his job in the U.S. prevented him going as frequently at the time of the interview.

[Interviewer: Have you ever gone with her, your wife, to this with the Pap test or these other check-ups?

Subject 6: Yes, when I have time, then, I go with her to the visit.

Interviewer: Have you ever gone with her to these visits?

Subject 7: Yes, I went with her once... before, I could go with her. Now I can’t. They don’t give me time.

Of the men who accompanied their partners, all but one reported that they went because of a mutual agreement with their female partner. Many of these men also reported that while they went to the office with their partners, they then remained in the waiting room for the duration of the appointment.

### Role of physician gender

Participants’ feelings regarding the gender of the gynecology provider seeing their partner were also explored. Almost all were agreeable to their partner seeing a male physician, although several felt that their partner would be more comfortable with a female physician.

[Interviewer: What do you think about that, that the doctor is a woman?

Subject 15: I think that… women understand each other better. It seems that it’s good, and … she likes that women see her.

Interviewer: Why does she prefer that?

Subject 15: She feels more confident.

Most felt that a physician of either gender would exhibit professionalism, so that they had minimal concerns regarding gender. However, multiple participants also mentioned that they were aware of abuse that had occurred between male physicians and female patients in their home countries. For some, this led them to encourage their partners to see female physicians. Although responses varied, it appeared that the motivation behind these statements may have been more fear of abuse than actual knowledge of specific incidents.

Interviewer: Do you think that the sex of the doctor, the gynecologist, is important for this type of thing?

Subject 17: It has a lot to do with a lot, because it’s confidential, first of all. Secondly, it’s not like a husband. We wouldn’t like for a male doctor to examine a woman. That’s why. Interviewer: You’d like it to be a woman? You maybe feel a little uncomfortable with a man?

Subject 17: Right.

Interviewer: Why?

Subject 17: Because there are going to be times when you don’t know if a doctor is doing their job or doing something bad that they shouldn’t.

The participants were also questioned regarding whether they would have a gender preference for a urologist, if they were to have a problem with their genitalia or sexual health; over half indicated that they would prefer a male urologist, again with most citing that they would be less embarrassed to discuss sexual health matters with another male.

Interviewer: If you ever had to check something about your genitals, would you go to a male or female doctor?

Subject 3: For me, I feel more like with a man than a woman.

Interviewer: Why?

Subject 3: Well, I don’t know, because I would have more communication with a male doctor.

To explore possible reasons behind male influence on cervical cancer screening, participants were asked whether they felt that women who went for frequent pelvic exams were promiscuous. None of the participants reported that they personally felt that way, but a majority reported that other men in their community may feel that way, and most reported a general poor perception within their community of women going to see male physicians.

Interviewer: There are some men that don’t like their wives to go to a male gynecologist... Why do you think that?

Subject 10: Ah, now that has to be machismo or jealousy... Many people will get like [that]... because their parents have taught them that or they didn’t have an education.

Reasons cited by participants included jealousy or insecurity of male partners, lack of education regarding the importance of gynecologic exams, concern for abuse by a male physician, and *machismo*. While only a few of the participants who responded stated that they feel their partner should ask their permission before seeing a doctor, almost all thought that other men would want their partner to ask permission.

Interviewer: There are some … many men that think that women should consult with their husbands before going to see a male gynecologist. What do you think about that?

Subject 3: I think so. Not for maybe trusting her, but rather, I think it’s part of communication between the couple.

## Discussion

A barrier to timely cervical cancer screening that has been infrequently assessed is the role of male partners and spouses. Male partners can have a great deal of influence on women’s healthcare choices and may affect their adherence to cancer screening [11]. Studies conducted in Africa have found that men often have little knowledge of the purpose of Pap smears and HPV screening. A survey of women undergoing HPV screening in western Kenya consistently demonstrated that even though most women felt that their own partners encouraged them to be screened for HPV-related disease, there was still a general sense of male partner distrust, a need to ask for male partner permission to attend a screening appointment, and a lack of male partner education regarding the importance of HPV screening in cervical cancer [24]. Similarly, a survey of men in Ghana showed that they admitted to little knowledge regarding cervical cancer, had many misconceptions about the cause of cervical cancer, and did not want their female partners going to health maintenance exams with male physicians [25]. Similar results regarding the knowledge of HPV and its role in the development of cervical cancer have also been reported among men in the U.S. [21]. However, contemporary attitudes and behaviors by which men influence the patterns of cervical cancer screening remain poorly documented.

Our current work builds upon previously studies of this topic in a number of important ways. Similar to prior reports both from the U.S. and abroad, we found that the male subjects interviewed for this study were generally aware that Pap smears exist and that they constitute an important part of women’s health care. However, fewer than half of the men interviewed were specifically aware of cervical cancer or could describe the role of Pap smears in preventing this disease. At the same time, misconceptions about Pap smears were commonly observed among many of our subjects. These observations are consistent with prior reports documenting knowledge gaps on this topic among men and emphasize the need to improve knowledge of HPV-related disease [11, 14, 18, 21, 25].

One of the key observations of our current work is that multiple subjects stated that they had obtained information regarding the Pap test at some point in their past either from literature at a doctor’s office or from television. None of the male participants in this survey reported having learned about HPV or HPV-related cancers from a community class or directly from a health professional. This observation is at least partially consistent with at least one prior report [11], which documented that Mexican immigrants in Multnomah County, Oregon, learned about Pap smears from multiple difference sources, including family members, health professionals, community classes, and media campaigns. The fact that at least some of our male subjects had taken time to learn more about Pap tests and cervical cancer suggests that male partners may be amenable to learning more about the impact of HPV infection on women and the specific nature of cervical cancer screening if provided an opportunity. For example, one of the subjects interviewed clearly describes having collected pamphlets at his significant other’s gynecologist and taking the time to actively read them. It will be important to better assess this issue across a broader sample of subjects in a more quantitative fashion in the future.

A second finding from our study is that many of the subjects stated that they accompany their wives to physicians’ visits without direct contact with the care provider. This observation is important for a number of reasons. First, many of the subjects interviewed stated that they waited for their female partners outside the examination room while their female partner was seen by her care provider. This potentially creates an opportunity that could be leveraged to better educate male spouses/partners. As several of our subjects indicated that some of their learning had taken place from media available at the physician’s office, a more intensive education outreach program within these offices could provide more comprehensive information about cervical cancer and HPV-related disease. Thus, it may be helpful to ascertain in the future if this represents a tractable opportunity for educating men about HPV, cervical cancer screening and, if so, what routes for learning would be preferred. Future work could focus on better assessing the answers to these questions in more precise and/or quantitative fashion then was possible in this study. One opportunity that could be leveraged in this regard could be the use of dyadic counseling [26]. Use of dyadic psychosocial interventions has been previously shown to benefit both members of adult couples coping with mental or physical health conditions [27]. These interventions appear to be particularly effective in situations such as the treatment of locoregionally advanced head and neck cancers, where both partners experience significant cognitive and emotional stress and may be having difficulty expressing emotional disclosures [28]. However, use of dyadic counseling in context of improving rates of cervical cancer screening has not yet been explored.

Another option that might help to improve knowledge of HPV and cervical cancer screening could be the use of patient navigators. Thompson et al. [16] have previously reported that using an intensive community outreach program with *promotoras*, or lay health workers with knowledge of a particular cultural community, was effective in increasing cervical cancer screening rates among Latinas in a rural eastern Washington community. Participants in the intensive group had significantly higher screening rates than women in usual care and low-intensity outreach (video education) groups. Shokar et al. [3] performed a community-wide, multidisciplinary intervention in El Paso County and the neighboring Hudspeth County to improve cervical cancer screening rates, including an educational and outreach component. After providing bilingual and culturally-directed education, they found that patients who received the intervention were 14 times more likely to participate successfully in screening. These studies have also documented that, given the language and cultural barriers that exist within immigrant Hispanic communities, strategies to increase screening found to be most effective are those that use a *promotora*, or a lay health person who is familiar with the languages and cultural nuances within the community [3, 16]. At present, it is not yet known whether use of *promotoras* or patient navigators would improve knowledge of HPV-related infections among male partners of women presenting for gynecologic care. However, it may be worthwhile to explore whether the focus of *promotora* programs for cervical cancer screening could be expanded to offer timely and effective solutions to address some of the barriers identified in this study.

A third important theme that emerged from our study is a sense of distrust for the health care system expressed by some subjects. Although participants cited multiple reasons for this distrust, many were related to prior experiences by the immigrants interviewed with health care providers in their home country. Several had either experienced or heard about instances of sexual abuse by a physician – particularly a male physician – or of infidelity. Overall, the sentiments of respondents were neutral with regard to their feelings about women going to see male gynecologists or going to a gynecologist for frequent cervical cancer screening. However, the respondents who accompanied their partners to the gynecologist most often did so by what they described as “mutual agreement” rather than their female partner’s request. Most of our study participants also admitted that they knew of other men within their communities who would look upon either frequent gynecologic exams or seeing a male gynecologist unfavorably, and that other men in their communities would require their female partners to ask permission prior to going to see a physician. Among reasons cited for this were jealousy and *machismo*, which were common themes among the answers. Further investigation is needed to assess the extent to which these attitudes and knowledge are present in the Hispanic immigrant population.

Strengths of the current study include its use of qualitative interviews to gain in-depth understanding of Hispanic immigrant men’s knowledge and attitudes toward cervical cancer screening. With the 19 interviews, we reached a point of saturation; i.e., a point where no new themes emerged with additional interviews. Other strengths include the diversity of recruitment sites representative of different subpopulations of the Hispanic immigrant population in the Houston, TX community, and recruitment of subjects from the community rather than a group from a health center that has more potential to introduce selection bias. Thus, the attitudes in the interviews may be more likely to reflect those found in that larger community, and possibly those of a more widespread Hispanic population. Limitations include the limited duration of the interviews and the lack of sociodemographic data collected from participants. A richer understanding of the context of men’s knowledge and attitudes could be achieved through a more comprehensive ethnographic approach, including the perspective from the female partners, which was not achieved through this study. We intend to address this deficit through the use of more quantitative work in which specific demographic features, religious beliefs and other potential behavioral drivers can be assessed in greater detail.

## Conclusion

Our current work has produced qualitative data from in-depth interviews with immigrant men and provides a richer understanding of their knowledge and attitudes toward cervical cancer screening. Hopefully, the insight generated here can be used in the future as a starting point for developing strategies to improve timely cervical cancer screening by overcoming barriers involving male partners.

## Data Availability

All primary data and research materials relevant to this manuscript are available from the corresponding author (MA) upon reasonable request in writing.

## Abbreviations

ACS: American Cancer Society
FDA: United States Food and Drug Administration
HPV: Human Papilloma Virus
HR HPV: High-Risk Human Papilloma Virus
IRB: Institutional Review Board
U.S.: United States
WHO: World Health Organization
